# Dietary Concentrate Supplementation Alters Serum Metabolic Profiles Related to Energy and Amino Acid Metabolism in Grazing Simmental Heifers

**DOI:** 10.3389/fvets.2021.743410

**Published:** 2021-10-26

**Authors:** Hao Chen, Chunjie Wang, Simujide Huasai, Aorigele Chen

**Affiliations:** ^1^College of Animal Science, Inner Mongolia Agricultural University, Hohhot, China; ^2^College of Veterinary Medicine, Inner Mongolia Agricultural University, Hohhot, China

**Keywords:** heifer, serum, growth performance, metabolic profiles, concentrate supplementation

## Abstract

Supplementation plays a vital role in the growth performance of grazing heifers. We investigated the effects of maize-based concentrate supplementation on the serum metabolome in grazing heifers. Twenty-four 7-month-old heifers (211.65 ± 4.25 kg BW) were randomly divided into a supplement (SUP) group and a control (CON) group. The results indicated that concentrate supplementation increased the final body weight (BW) of grazing heifers, and the average daily gain (ADG) was 61.5% (*P* = 0.011) higher in the SUP group than in the CON group. Serum concentrations of total protein (TP), triglyceride (TG), and leptin were higher in the SUP group than in the CON group (*p* < 0.05). Supplementation increased serum metabolites and amino acids and markedly altered glucose, lipid, and protein metabolism, which contributed to the heifer growth. Furthermore, by multivariate analysis, 45 serum metabolites were identified as significantly different between the two groups. Enrichment analysis revealed that arginine biosynthesis and tryptophan metabolism as well as glycerophospholipid metabolism were significantly enriched between the two groups. We concluded that the growth potential of heifers could be improved by maize-based concentrate supplementation, and the main biological pathways affected were those related to energy and amino acid metabolism.

## Introduction

Grazing is a very important production system for the ruminant industry worldwide due to its low cost and benefit to animal welfare (1). A major challenge of this system is the imbalance between pasture availability and animal nutritional requirements (2). Most beef cattle are grazed extensively on pasture without any feed supplements on the grasslands of Inner Mongolia in northern China, which results in decreased animal performance and low productivity (3). Many researchers have revealed that the dry matter intake (DMI) and productivity of grazing ruminants during periods of pasture grazing or nutrient deficiencies could be improved by supplementation with protein (4), minerals (5), or multinutrient concentrates (6). Pasture availability and nutritive value seem to be sufficient to meet the requirements for cattle grazing in summer. However, according to Li et al. (7) and preliminary research by our team (8), Simmental cows at various growth stages with concentrate supplementation showed better growth performance than those not receiving supplementation. Therefore, the growth potential of grazing cattle may be further exploited by supplementing concentrate in summer. In addition, supplementation could help alleviate the pressure of grassland degradation caused by overdependence on the grasslands of Inner Mongolia (9).

To date, animal performance has been compared in steers (10), beef cows (11), and calves (12) under grazing and supplementation conditions, mainly reflecting a mixed effect of feed type and amounts. However, few studies have compared the growth performance in heifers by supplementing concentrate until now. Previous research has shown that heifers could achieve breeding size and maturity relatively early by improving the nutrient level, which potentially reduces the rearing costs of replacement heifers (13). Additionally, the metabolic mechanisms by which concentrate supplementation improves growth performance are insufficiently understood.

Metabolomics has become an emerging research area that quantitatively measures changes in all small-molecule metabolites in biological samples caused by changes in nutrient levels and therefore it can directly reveal changes in the metabolic state of organisms (14). Many studies have examined the effects of dietary composition on changes in metabolic states. For example, Leal et al. (15) found that dairy Holstein calves fed different pre-weaning nutrient diets influenced protein and energy metabolism. A recent study suggested that supplementation with highland barley is involved in regulating the metabolism of several lipid-related metabolites (16). Liquid chromatography-tandem mass spectrometry (LC-MS/MS) has been widely used for the identification of biomarkers and metabolism pathway characterization in cows due to its high resolution, detection sensitivity and non-derivatization of samples (17).

In the present study, we aimed to investigate the changes in metabolic phenotypes of heifers following concentrate supplementation. Then, we used metabolomics profiling in conjunction with growth parameters to identify the main metabolic pathways associated with greater growth in heifers supplied concentrate and to provide a theoretical basis for heifer supplementary feeding.

## Methods

### Animals, Diets, and Feeding Regimes

Twenty-four 7-month-old Simmental heifers (211.65 ± 4.25 kg BW) were randomly divided into a supplement (SUP) group and a control (CON) group (*n* = 12 per group). The heifers in the CON group were grazed on a *Leymus chinensis*-based pasture without any supplementation, and those in the SUP group were grazed on the same grassland but received a concentrate supplement. All the heifers were released to graze during the daytime for the 60-day trial; from d 1 to d 30, the heifers in the SUP group were housed and individually received supplementary concentrate at a rate of 1.12 kg of DM/heifer per day (1.2 kg of fresh weight/heifer per day), from d 31 to d 60, the heifers in the SUP group were housed and individually received supplementary concentrate at a rate of 1.44 kg of DM/heifer per day (1.6 kg of fresh weight/heifer per day) when returned to the enclosure after grazing. We observed that the animals consumed all the concentrate offered; that is, no refusals were recorded. The ration compositions and nutrient levels of the concentrate are listed in Table 1. All the heifers had free access to water throughout the experiment. The duration of the experiment was 68 days, and the heifers were offered the experimental diets for an 8-d dietary acclimatization period. Following this period, the animals remained on their treatments for an additional 60 days.

**Table 1:** Composition of concentrate fed to Simmental heifers.

**Ingredient**	**Composition (%)**
Maize	47.75
Wheat bran	25.25
Soybean meal	21.00
Limestone	2.0
CaHPO4	1.0
NaCl	1.0
Premix	2.0
Total %	100
Fraction (% DM)	
Dry matter (%)	91.36
Crude protein	18.22
Neutral detergent fiber (NDF)	21.63
Acid detergent fiber (ADF)	14.88
Ether extract (EE)	3.19
ME (MJ/kg)	13.15

The DM, gross energy, ether extract, and crude protein were analyzed according to the AOAC (18), and acid detergent fiber (ADF) and neutral detergent fiber (NDF) were analyzed according to Van Soest et al. (19). Concentrate samples were collected weekly and pooled for nutritional analysis. The BW data of each heifer were recorded on days 1 and 60 during the formal trial using an electronic scale. Average daily gain (ADG) was calculated as ADG = (Final BW-Initial BW)/60 (kg/day).

### Blood Sampling

Blood samples were collected from the jugular vein into evacuated tubes on the first and last days of the trial between 0600 and 0800 h before grazing in the morning. The samples were centrifuged at 3,000 rpm and 4°C for 10 min to obtain serum samples, immediately transferred to the laboratory and stored at −80°C. Seven serum samples were randomly selected from each group and prepared for further analysis.

### Analysis of Serum

Enzyme-linked immunosorbent assay (ELISA) methods were used to determine serum triglyceride (TG), leptin, total protein (TP), albumin (ALB), and cholesterol (CHO) (Sino-UK Institute of Biological Technology, Beijing, China) concentrations.

### Metabolite Extraction and Quality Control

Two hundred microliters of serum sample was accurately weighed, and the metabolites were extracted using 400 μL of methanol:water (4:1, v/v) solution. The mixture was allowed to settle at −20°C and treated with a Wonbio-96c high-throughput tissue crusher (Shanghai Wanbo Biotechnology Co., Ltd.) at 50 Hz for 6 min, followed by vortexing for 30 s and ultrasonication at 40 kHz for 30 min at 5°C. The samples were stored at −20°C for 30 min to precipitate proteins.

After centrifugation at 13,000 g at 4°C for 15 min, the supernatant was carefully transferred to sample vials for LC-MS/MS analysis (20). As a part of the system conditioning and quality control (QC) process, a pooled QC sample was prepared by mixing equal volumes of all samples. QC samples were prepared and tested in the same manner as the analytical samples. The QC samples helped represent the whole sample set and were injected at regular intervals (every 5 samples) to monitor the stability of the analytical instrument.

### UHPLC-Q-TOF-MS/MS Analysis

Chromatographic separation of the metabolites was performed on a Thermo UHPLC system equipped with an ACQUITY BEH C18 column (100 × 2.1 mm i.d., 1.7 μm; Waters, Milford, USA). The mobile phases consisted of 0.1% formic acid in water (solvent A) and 0.1% formic acid in acetonitrile:isopropanol (1:1, v/v) (solvent B). The solvent gradient program was as follows: 0 to 3 min, 95:5 to 80:20 A/B; 3 to 9 min, 80:20 to 5:95 A/B; 9 to 13 min, 5:95 A/B; 13 to 13.1 min, 5:95 to 95:5 A/B; and 13.1 to 16 min, 95:5 A/B for system equilibration. The sample injection volume was 2 μL, and the flow rate was 0.4 mL/min. The column temperature was maintained at 40°C. During analysis, all samples were stored at 4°C.

The mass spectrometric data were collected using a Thermo UHPLC-Q Exactive mass spectrometer equipped with an electrospray ionization (ESI) source operating in either positive or negative ion mode (POS and NEG, respectively). The optimal conditions were set as follows: Aus gas heater temperature, 400°C; sheath gas flow rate, 40 psi; Aus gas flow rate, 30 psi; ion-spray voltage floating (ISVF), −2,800 V (NEG) and 3,500 V (POS); and normalized collision energy, 20–40–60 V rolling for MS/MS analysis. Data acquisition was performed in data-dependent acquisition (DDA) mode over a mass-to-charge (m/z) ratio range of 70–1,050.

### Data Pre-processing and Annotation

After ultrahigh-performance liquid chromatography-tandem quadrupole time of flight mass spectrometry (UHPLC-Q-TOF-MS/MS) analyses, raw data were imported into Progenesis QI 2.3 (Non-linear Dynamics, Waters, USA) for peak detection and alignment. The pre-processing results generated a data matrix that consisted of the retention time (RT), m/z, and peak intensity values (21). Metabolic features with at least 80% detection in any set of samples were retained. After filtering, minimum metabolite values were imputed for specific samples in which the metabolite levels fell below the lower limit of quantitation, and each metabolic feature was normalized by the sum. An internal standard was used for data QC (reproducibility). Metabolic features with a relative standard deviation (RSD) of QC > 50% were discarded (20). Mass spectra of these metabolic features were identified by using accurate mass spectrometry, MS/MS fragment spectral and isotope ratio differences, and searching reliable biochemical databases, such as the Human Metabolome Database (HMDB) (http://www.hmdb.ca/) and Metlin database (https://metlin.scripps.edu/). Specifically, the mass tolerance between the measured m/z values and the exact mass of the components of interest was ±10 ppm. For metabolites with MS/MS confirmation, only those with MS/MS fragment scores above 30 were considered confidently identified. Otherwise, metabolites had only tentative assignments.

### Statistical Analysis

Performance measurements such as BW, ADG, DMI, and serum parameters were performed using the one-way ANOVA procedure of SPSS statistical software (Version 17.0; SPSS Inc. Chicago, IL, USA). Duncan's multiple range test was used to compare the differences among the treatment groups. The data are expressed as the mean ± SEM. *P* < 0.05 was used as the minimum level of significance.

Multivariate statistical analysis was performed using the ropls (Version 1.6.2, http://bioconductor.org/packages/release/bioc/html/ropls.html) R package from Bioconductor on the Majorbio Cloud Platform (https://cloud.majorbio.com). Principal component analysis (PCA) using an unsupervised method was applied to obtain an overview of the metabolic data, and general clustering, trends, or outliers were visualized. All of the metabolite variables were scaled to unit-variances prior to conducting PCA (22). Orthogonal partial least squares discriminate analysis (OPLS-DA) was used for statistical analysis to determine global metabolic changes between comparable groups (23). All of the metabolite variables were subjected to Pareto scaling prior to conducting OPLS-DA. The model validity was evaluated from model parameters R2 and Q2, which provide information for the interpretability and predictability, respectively, of the model and avoid the risk of overfitting (21). Variable importance in the projection (VIP) values were calculated in the OPLS-DA model. The *p*-values were estimated with paired Student's *t*-test in single-dimensional statistical analysis (24). Statistically significant differences among groups were selected with VI *P* > 1 and *p* < 0.05 (25). Differential metabolites between the two groups were summarized and mapped into their biochemical pathways through metabolic enrichment and pathway analysis based on a database search (KEGG, http://www.genome.jp/kegg/) (26). The Stats package in R and the SciPy package in PYTHON (https://docs.scipy.org/doc/scipy/) were utilized to identify significantly enriched pathways using Fisher's exact test (27).

## Results

### Growth Performance

The effects of concentrate supplementation on the growth performance of grazing heifers are presented in Table 2. The initial BW did not differ between the SUP and CON groups, but after 60 days, a significant difference in the final BW (*p* < 0.05) was found between the two groups, and the ADG was 61.5% (*p* < 0.05) higher in the SUP group than in the CON group.

**Table 2:** Effects of concentrate supplementation on body weight and blood indices of grazing heifers.

**Items**	**CON**	**SUP**	***P*-value**
Body weight (kg)			
Day 1 of the trial	217.33 ± 5.19	215.01 ± 4.68	0.632
Day 60 of the trial	245.53 ± 6.52	260.55 ± 7.86	0.027
ADG (kg)	0.470 ± 0.083	0.759 ± 0.132	0.011
Triglyceride (mmol/l)			
Day 1 of the trial	0.36 ± 0.04	0.33 ± 0.02	0.317
Day 60 of the trial	0.41 ± 0.02b	0.52 ± 0.05a	0.016
CHO (mmol/l)			
Day 1 of the trial	7.66 ± 0.32	7.18 ± 0.19	0.412
Day 60 of the trial	7.85 ± 0.65	10.12 ± 0.373	0.022
TP (mg/ml)			
Day 1 of the trial	117.26 ± 10.51	121.58 ± 12.69	0.262
Day 60 of the trial	123.50 ± 16.52	147.98 ± 19.11	0.001
ALB (mg/ml)			
Day 1 of the trial	57.73 ± 4.57	58.02 ± 5.66	0.158
Day 60 of the trial	58.40 ± 6.29	69.63 ± 8.32	0.071
Leptin (ng/ml)			
Day 1 of the trial	5.10 ± 0.43	5.29 ± 0.65	0.671
Day 60 of the trial	5.78 ± 0.55b	7.19 ± 0.81a	0.001

*^a, b^ Means bearing different superscripts in the same row differ significantly (P <0.05)*.

### Serum Biochemical Parameters

Concentrations of biochemical parameters in serum on days 0 and 60 are presented in Table 2. On day 0 of the experiment, the differences in blood indices between groups were not significant (*p* > 0.05). Serum concentrations of TP, TG, and leptin were higher in the SUP group than in the CON group on day 60 (*p* < 0.05). The CHO and ALB concentrations on day 60 between groups were not significantly different (*p* > 0.05).

### Multivariate Analysis of Serum Metabolites

**Figure 1:**
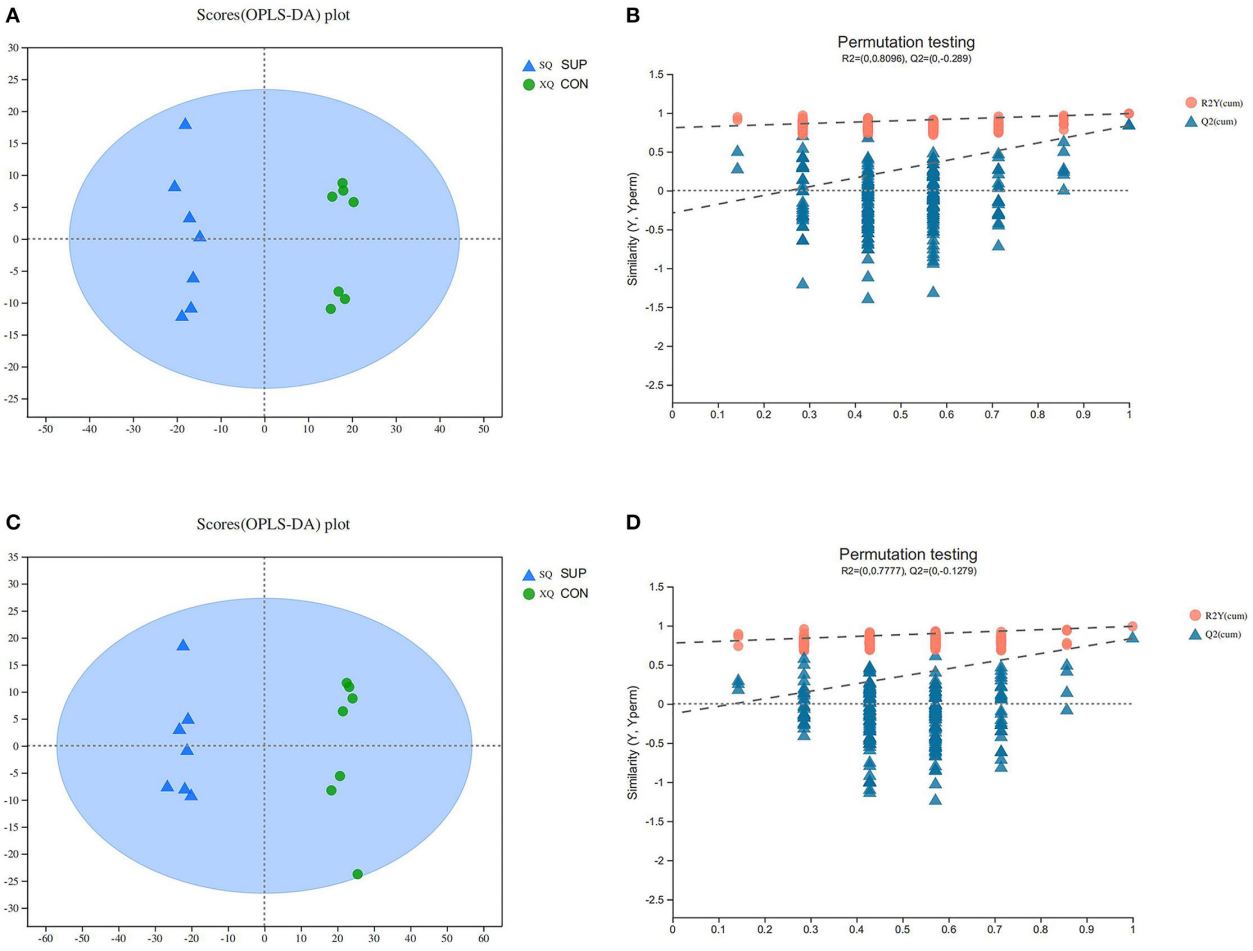
Corresponding validation plots of orthogonal projections to latent structures-discriminate analysis (OPLS-DA) and permutation test of the OPLS-DA model **(B,D)** were derived from the UHPLC-QTOF/MS metabolomics profiles of serum. **(A,B)** and **(C,D)** were respectively, derived from POS and NEG for the serum samples. Blue and green respectively, represent SUP and CON administration to heifers. SUP, supplementation heifers; CON, grazing heifers. *n* = 7.

UHPLC-Q-TOF-MS/MS was conducted with POS and NEG ESI. QC samples were analyzed after every five samples to determine the stability and repeatability of the system. Supplementary Figure 1 shows the overlap of the total ion chromatograms of the QC sample in POS (A) and NEG (B). The results confirmed the reliable repeatability and precision of the data obtained in this study. Score plots of the (O)PLS-DA performed to verify the different metabolites between the two groups and supervise the multivariate analysis are shown in Figures 1A,C. All the heifer serum samples were within the 95% Hotelling T2 ellipse in the score plots. The validity of the OPLS-DA model was evaluated using R2Y and Q2 values. In this study, the R2Y values of the POS and NEG serum samples were 0.993 and 0.991, respectively.

**Figure 2:**
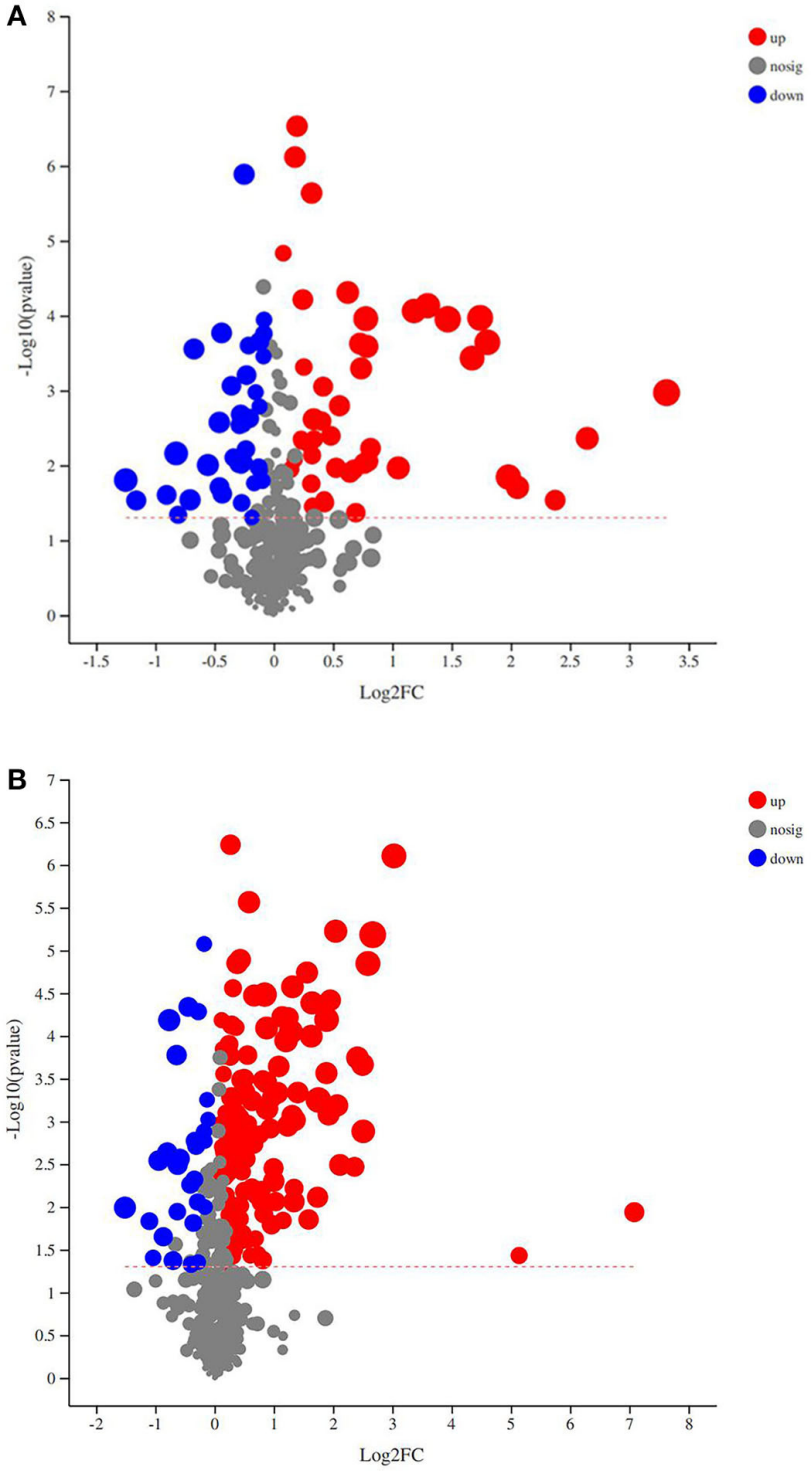
**(A,B)** were respectively, derived from POS and NEG of the serum samples. In the volcano plot, each point represents a metabolite, and the point size represents the VIP value of this metabolite in the OPLS-DA model. Compared with the CON group, red indicates a significantly upregulated metabolite in the SUP group, whereas blue indicates the opposite, and gray shows no significant difference between the two groups.

To estimate the robustness and predictive ability of our models, we used the 7-fold cross validation method with permutations, and Q2 intercept values were obtained after 200 permutations; the results are shown in Figures 1B,D. The permutation test results for the Q2 intercepts were −0.28 for POS serum and −0.12 for NEG serum samples. Both POS and NEG data revealed clear separation and discrimination between the CON and SUP groups, indicating that the (O)PLS-DA model can be used to identify differences between the two groups. With a *t*-test *p* < 0.05 and a OPLS-DA model VIP threshold of 1 as criteria, significantly differential metabolites between the two groups were screened from all identified metabolites. The significantly differential metabolites were visualized through volcano plots (Figure 2). The figure clearly shows that many metabolites in the serum samples were significantly different between the two groups.

### Significantly Differential Metabolites Between the Two Groups

As shown in Table 3, among the 45 significantly differential metabolites in the serum, 26 metabolites had higher concentrations in the SUP heifers than in the CON heifers (Table 3). The concentrations of mostly identified amino acids, including tyrosine (FC = 1.647), tryptophan (FC = 1.586), glutamine (FC = 1.669), glycine (FC = 2.651), asparagine (FC = 4.240), leucine (FC = 2.671), arginine (FC = 2.942), and lysine (FC =2.848), increased in the SUP heifers compared to the CON heifers. The succinate (FC =3.501), α-ketoglutarate (FC = 1.559), citrate (FC = 1.370), oleic acid (FC = 2.004), and pregnenolone (FC = 1.591) concentrations were higher in the SUP heifers than in the CON heifers.

**Table 3:** Identification of significant differential metabolites in heifer serum by comparison of the concentrate and forage groups using a VIP threshold of 1 (*P* < 0.05).

**Metabolite**	**VIP**	**FC**	**M/Z**	***P*-value**	**RT**
6-(3-carboxypropoxy)-3,4,5-trihydroxyo xane-2-carboxylic acid	2.054	4.383	301.05	0.036	2.01
Cinncassiol D4 2-glucoside	2.937	12.196	578.29	0.001	3.89
Oleic acid	1.836	2.004	183.10	0.006	5.76
Ganoderenic acid A	1.728	2.934	549.26	0.005	6.99
Tocopheronic acid	2.488	0.485	339.14	0.001	5.51
2-hydroxyhexadecanoic acid	1.081	0.925	271.23	0.001	9.06
Soyacerebroside I	1.103	1.131	734.52	0.004	11.18
Dihydrocortisol	1.385	1.338	363.21	0.031	7.86
PE(15:0/18:0)	1.231	0.909	750.52	0.003	10.97
PE[15:0/20:1(11Z)]	1.074	0.929	776.53	0.005	11.01
PE[15:0/18:2(9Z,12Z)]	1.471	0.826	746.49	0.004	10.17
MG(0:0/15:0/0:0)	1.389	0.812	631.47	0.007	10.10
PC[20:1(9Z)/0:0]	1.398	0.941	550.38	0.025	9.13
PE[18:3(6Z,9Z, 12Z)/P-18:0]	1.243	1.130	770.52	0.013	10.64
PC(14:0/0:0)	1.689	0.917	468.31	0.016	7.75
PS[20:0/20:3(5Z,8Z,11Z)]	1.104	1.110	840.56	0.002	11.01
Digoxigenin bisdigitoxoside	1.857	0.521	649.36	0.036	6.53
Galactosylhydroxylysine	1.593	2.099	369.15	0.001	6.73
Tyrosine	1.798	1.647	198.08	0.005	4.95
Tryptophan	1.341	1.586	293.16	0.039	2.91
Glutamine	1.679	1.669	363.15	0.026	1.04
Arginyl-Gamma-glutamate	1.201	1.788	337.14	0.024	2.97
N-Hydroxy-L-tyrosine	1.827	0.648	198.07	0.031	4.95
N-Decanoylglycine	1.734	0.684	228.15	0.002	6.65
Glycine	1.292	2.651	144.06	0.036	0.82
Asparagine	2.331	4.240	295.11	0.043	5.33
Leucine	1.237	2.671	665.23	0.025	4.98
Arginine	1.034	2.942	261.14	0.001	0.77
Gamma-Glutamylvaline	1.188	0.939	247.13	0.001	1.76
Lysine	1.990	2.848	517.30	0.003	7.27
Isomugineic acid	1.305	1.802	365.12	0.042	4.23
Hydrocinnamic acid	1.806	0.497	149.06	0.001	5.01
1,4-Dihydroxy-2-naphthoic acid	1.479	0.616	203.03	0.001	5.62
Sphingosine 1-phosphate	1.552	1.326	268.33	0.035	6.85
α-ketoglutarate	1.812	1.559	514.21	0.013	7.15
Pregnenolone	2.048	1.591	677.48	0.001	9.89
Hippuric acid	1.840	0.819	180.06	0.015	3.20
O-Desmethylcarvedilol	2.194	0.675	437.17	0.001	3.88
Citrate	2.658	1.370	257.05	0.032	5.98
Alpha-CEHC glucuronide	2.105	2.194	487.21	0.033	3.41
N-Monodesmethyl-rizatriptan	2.848	0.642	303.19	0.038	4.01
2-(2-Furanyl)-3-methyl-2-butenal	2.036	1.450	133.06	0.001	5.02
Succinate	2.534	3.501	107.04	0.001	3.12
Coriandrone C	2.725	0.315	282.99	0.013	5.43
Lactic acid	1.823	0.762	245.03	0.001	6.65

*RT, retention time; fold change: SUP group vs. CON group*.

### Metabolic Pathway Analysis of Metabolites

**Figure 3:**
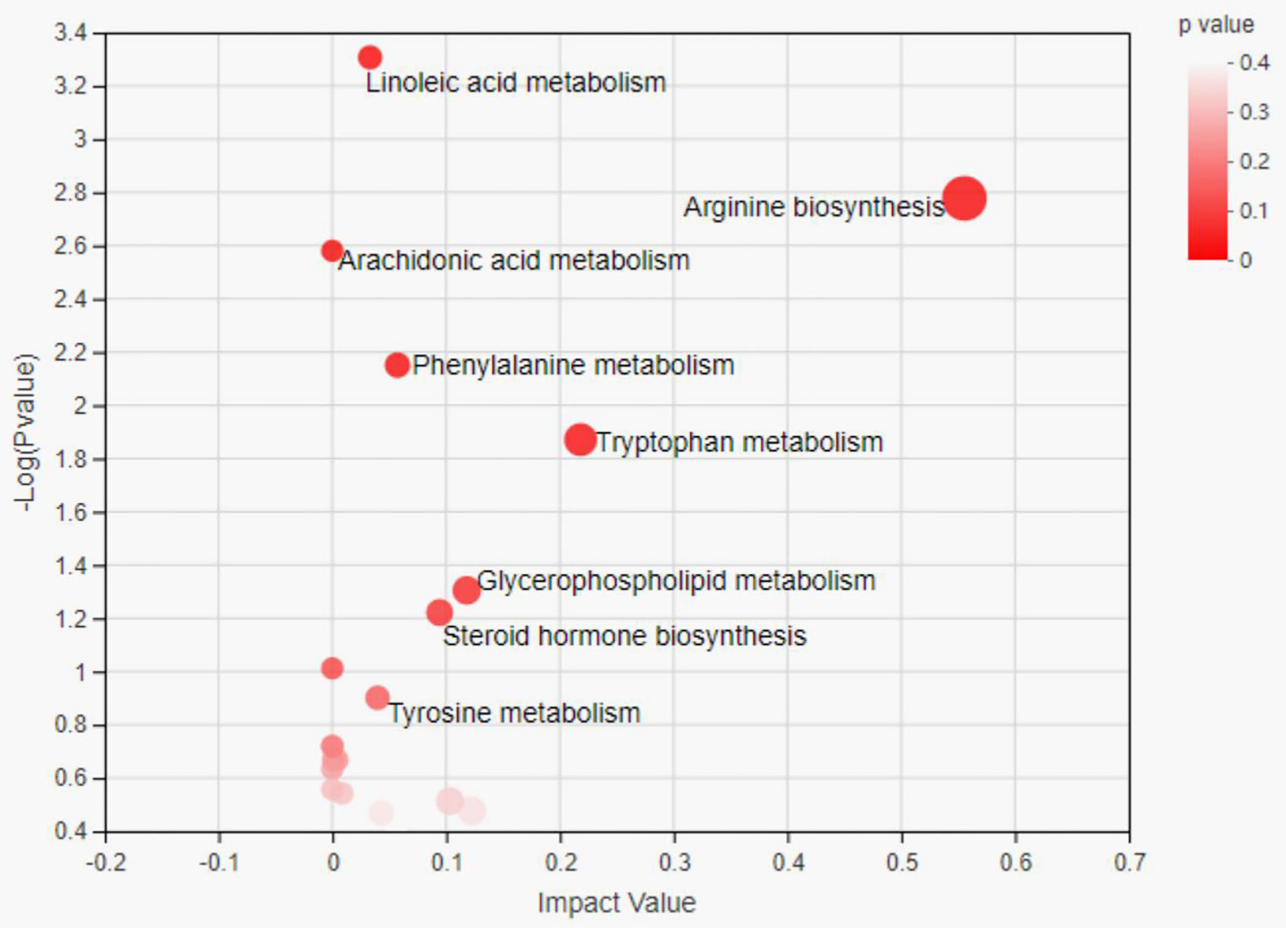
Pathway analysis of differential metabolites in serum samples between the two group heifers. Each bubble in the bubble map represents a metabolic pathway. The x-axis represents a pathway impact value in the topology analysis, and larger bubbles represent higher pathway impact values. The y-axis represents the *P*-value (–Log *p*) of the metabolic pathway in the enrichment analysis, and the darker color of bubble represents higher pathway enrichment.

The metabolome view map revealed the enriched pathways (*P* < 0.05) for metabolites that were identified in serum (Figure 3), along with pathway impact values in some cases. Enrichment analysis revealed that arginine biosynthesis and tryptophan metabolism as well as glycerophospholipid metabolism were significantly enriched in the SUP group compared to the CON group (Figure 3).

## Discussion

Higher ADG and final BW values in the heifers from the SUP group than in those from the CON group were recorded, which was in accordance with other studies concerning concentrate supplementation of growing cows in a summer pasture (4). These results suggest that concentrate supplementation could be effective in improving the performance of grazing heifers. Many results have been reported in different breed cattle, in which a significant increase in ADG was observed with an increase in dietary energy level (28, 29). Furthermore, it is necessary to supplement cattle with protein to maximize the nitrogen in the rumen and result in a greater ADG (30). In the present study, the dietary protein and energy levels were increased by supplying a concentrate, which resulted in higher ADG values. Therefore, supplementary feeding of concentrates is a promising strategy to improve the growth performance of grazing heifers and promote early breeding size and maturity, which potentially reduces the rearing costs of replacement heifers.

The leptin concentration in serum was positively correlated with the manifestation of fat deposition (31). In this study, there was a positive effect of concentrate supplementation on the serum concentration of leptin, indicating that concentrate supplementation increased the fat deposition of grazing heifers. The leptin level in yaks was positively correlated with ADG (32), suggesting that concentrate supplementation could promote the secretion of leptin and thereby increase growth, which is consistent with the results of this study. The value of serum TP is related to the physiological conditions of animals and the quality of protein contained in the diet (33). Under normal physiological conditions, an elevation in TP levels in blood is associated with better dietary protein nutrition (34). In this study, the serum TP concentration in the SUP group was significantly higher than that in the CON group, which could be related to the appreciably high protein intake from the supplied concentrate.

In the present study, metabolome data revealed that supplementary feeding altered the concentrations of serum metabolites. We found that the levels of numerous amino acids (tyrosine, glutamine, glycine, asparagine, leucine, arginine, and tryptophan) in serum were higher in the SUP group than in the CON group. Amino acids are important substances in tissue protein synthesis.

Glutamine serves as a major amino acid for promoting the synthesis of polypeptides and proteins and also functions as a vital regulator for reducing the decomposition of proteins (35). It has been reported that serum glutamine levels are increased by hull-less barley supplementation (31). Leucine mainly serves as a substrate for protein synthesis and is widely used in the estimation of protein synthesis in heifers (36). Furthermore, a recent study demonstrated that leucine could also act as a signal to increase the rate of protein synthesis in the skeletal muscle of calves (37). In this study, concentrate supplementation increased the concentrations of glutamine and leucine, indicating that supply feeding can influence protein metabolism. Our results revealed that the ADG from the SUP group was higher than that from the CON group, which may result from the increased serum levels of glutamine and leucine. Arginine is known to be a substrate for the synthesis of nitric oxide (NO), which can activate soluble guanylyl cyclase to promote vasodilation and induce insulin release (38). Arginine has also been proven to participate in the TCA cycle by affecting the formation of fumarate (39). In this study, concentrate supplementation increased the concentration of arginine, indicating that concentrate supplementation can influence protein and energy metabolism. Additionally, Jiao et al. (40) indicated that arginine in the urea cycle is related to the synthesis of antioxidant and anti-inflammatory molecules. In this study, arginine concentration was higher in the SUP group than in the CON group, indicating that concentrate supplementation may modulate oxidative stress. Based on our previous report, compared with heifers without any concentrate supply, supplement-fed heifers showed higher levels of superoxide dismutase and total antioxidant capacity and lower levels of malondialdehyde (41). As antioxidant compounds (vitamins, Cu, and Zn) were added to concentrate diets, altered metabolism or antioxidant compounds related to the changed oxidative status need to be proven by further research. Glycine is the prime metabolic source of glutathione, creatine, and purines, playing a vital role in various biological processes. In this study, we found a higher concentration of glycine in the CON group than in the SUP group.

Tyrosine is an important amino acid that comprises proteins, peptides and enkephalins, and it is also a precursor of thyroid hormone (42). In addition to being a substrate for protein synthesis, tryptophan can also be used as a signal molecule to participate in the regulation of the protein synthesis rate (43). Jansman et al. found a close positive correlation between the tryptophan concentration and the rate of protein synthesis (44). It has been reported that Trp can effectively promote the combination of ribosomes and mRNA and promote the formation of mRNA polyribosome complexes; tryptophan can increase the synthesis of energy by stimulating the secretion of insulin and by activating translation initiation factors (and eIF2α, phosphorylation level, eIF2B activity, and GTP binding to the initiation factor are related) to initiate translation (45). In this study, both tyrosine and tryptophan levels were higher in the serum of the SUP group than in that of the CON group, indicating sufficient substrate levels for protein synthesis and synthesis rate in the SUP group. This is consistent with the increases in BW and the concentration of serum TP of the heifers in the SUP group.

Notably, we observed that some of the changed metabolites, such as glycine, arginine and glutamine, can be used as glucogenic substrates. Gluconeogenesis generates glucose from these glucogenic amino acids, providing up to 90% of the glucose required for host maintenance and production in ruminants (46). Propionate is the predominant substrate for gluconeogenesis. Many researchers have indicated that the ruminal content of propionate increases with increasing amounts of dietary concentrate (47, 48). Therefore, it is reasonable to infer that a propionate-induced abundance of precursors for gluconeogenesis can allow these glucogenic amino acids to be utilized predominantly in protein synthesis.

Both succinate and citrate have been identified as key players in the TCA cycle, and α-ketoglutarate plays an important role in cellular energy metabolism by acting as a rate-determining intermediate in the TCA cycle (49). Some studies demonstrated that the levels of α-ketoglutarate in heifers were increased by feeding high levels of concentrate (47). In this study, the increased concentrations of succinate, citra and α-ketoglutarate may not only suggest the increased availability of substrate but also promote the TCA cycle. This study not only identified differential metabolites between the SUP and CON groups but also analyzed the metabolic pathways in which these metabolites were involved. In this study, we explored the key metabolic pathways based on the impact values and *p*-values. Through the analysis of metabolic pathways in serum, we found that the major metabolic pathways of the serum were arginine biosynthesis and tryptophan metabolism as well as glycerophospholipid metabolism.

## Conclusions

In conclusion, we used a metabolomics approach to analyze serum samples for evidence of alterations in key biological processes in grazing heifers supplied with maize-based concentrate. The growth potential of heifers could be improved by maize-based concentrate supplementation. Based on serum metabolomics analysis, we concluded that the main biological pathways affected were those related to energy and amino acid metabolism. Combined with the results of serum biochemical parameters, supplementation with maize-based concentrate could significantly improve energy and protein utilization efficiency compared to grazing heifers.

## Data Availability Statement

The original contributions presented in the study are included in the article/Supplementary Material, further inquiries can be directed to the corresponding author.

## Ethics Statement

The animal study was reviewed and approved by Institutional Animal Care and Use Committee in the College of Animal Science, Inner Mongolia Agricultural University, China. Written informed consent was obtained from the owners for the participation of their animals in this study.

## Author Contributions

HC wrote the main manuscript and prepared Figures 1–3. SH prepared Supplementary Figure 1. AC and CW editing the manuscript. All authors have read and agreed to the published version of the manuscript.

## Funding

This work was funded by the National Key R&D Program of China (2018 YFD0501700) and National Natural Science Foundation of China (project no. 31660677).

## Conflict of Interest

The authors declare that the research was conducted in the absence of any commercial or financial relationships that could be construed as a potential conflict of interest.

## Publisher's Note

All claims expressed in this article are solely those of the authors and do not necessarily represent those of their affiliated organizations, or those of the publisher, the editors and the reviewers. Any product that may be evaluated in this article, or claim that may be made by its manufacturer, is not guaranteed or endorsed by the publisher.
