# Supplementary figures and images for: Dietary Concentrate Supplementation Alters Serum Metabolic Profiles Related to Energy and Amino Acid Metabolism in Grazing Simmental Heifers
Source: Front Vet Sci. 2021 Oct 26;8:743410. doi: 10.3389/fvets.2021.743410 (PMC8577580; doi:10.3389/fvets.2021.743410)

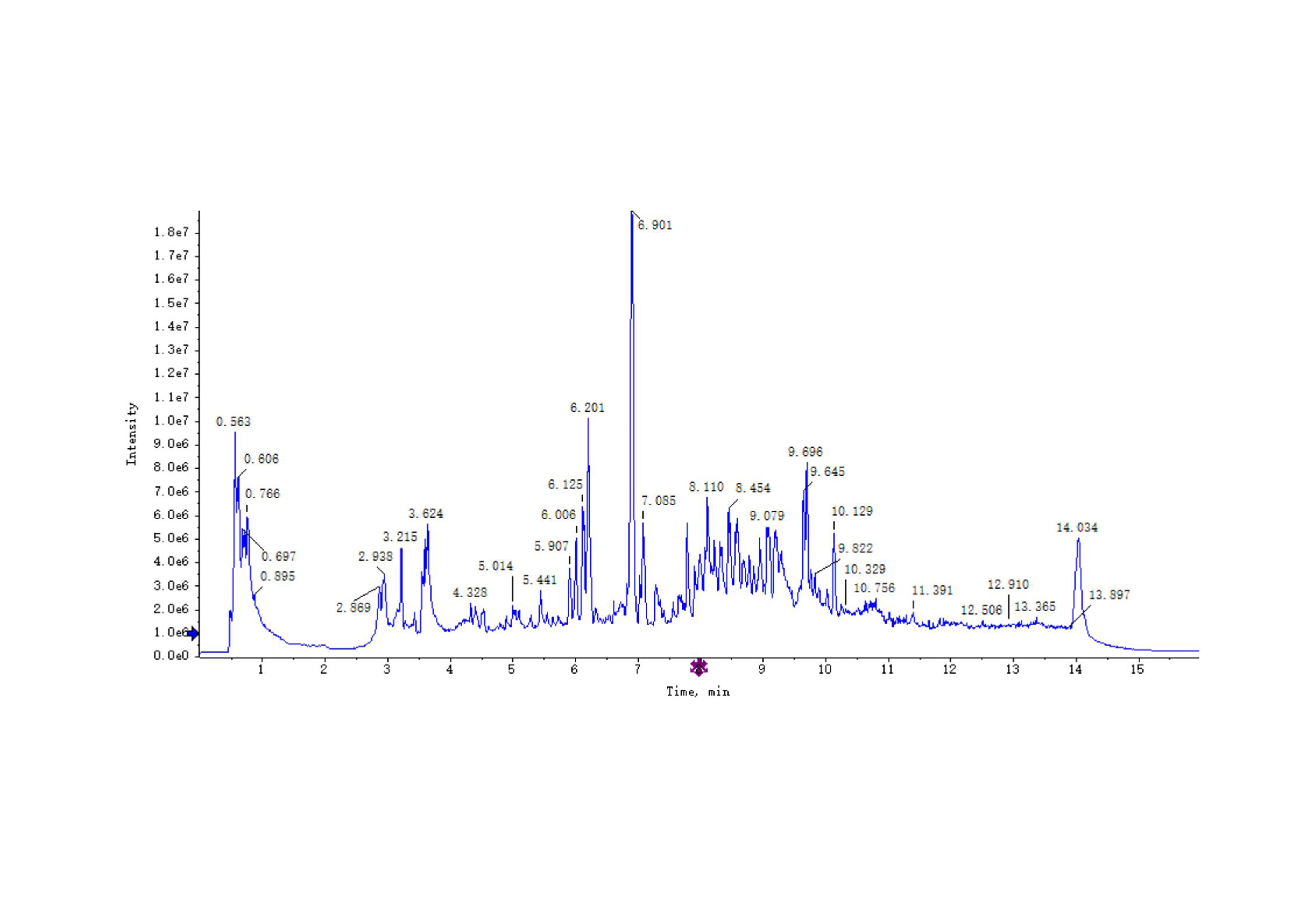

Supplement: Supplementary file 1 [file Image_1.JPEG]

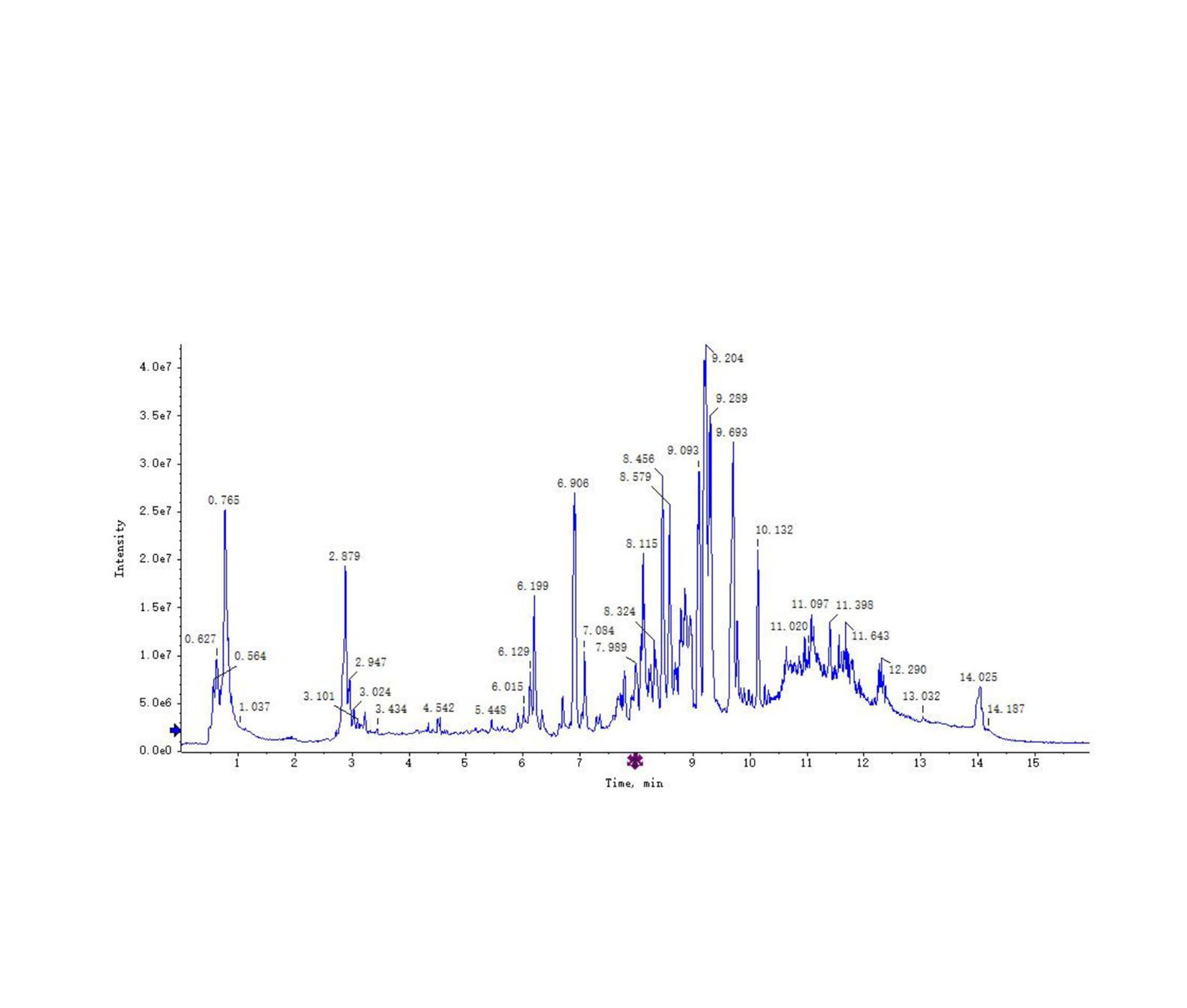

Supplement: Supplementary file 2 [file Image_2.JPEG]
